# Effects of Captivity and Season on the Gut Microbiota of the Brown Frog (*Rana dybowskii*)

**DOI:** 10.3389/fmicb.2019.01912

**Published:** 2019-08-23

**Authors:** Qing Tong, Xiao-Ning Liu, Zong-Fu Hu, Jia-Feng Ding, Jia Bie, Hong-Bin Wang, Jian-Tao Zhang

**Affiliations:** College of Veterinary Medicine, Northeast Agricultural University, Harbin, China

**Keywords:** microbial diversity, intestinal microbiota, red-leg syndrome, amphibian, 16S rRNA gene, ontogenetic

## Abstract

The gut microbiota of amphibians is affected by exogenous and endogenous factors. We performed a comprehensive analysis using high-throughput sequencing technology and functional predictions and observed general changes in the gut microbiota of frogs in different growth stages, seasons, and growth environments. There were no significant differences in microbial richness and diversity between juvenile and adult wild frogs, between the summer and autumn groups of captive frogs, or between wild and captive frogs. There were significant differences in the gut microbiota community structure of *Rana dybowskii* between the summer and autumn groups of captive frogs and between wild and captive *R. dybowskii*, whereas the differences between juvenile and adult wild frogs were not significant. The dominant gut bacterial phyla in frogs from both captive and wild environments included Firmicutes, Bacteroidetes, Proteobacteria, and Actinobacteria. Linear discriminant effect size (LEfSe) analysis showed that Bacteroidetes and Firmicutes were significantly enriched in captive and wild *R. dybowskii*, respectively linear discriminant analysis (LDA > 4). The core operational taxonomical units (OTUs) that were found in >90% of all frogs tested encompassed 15 core OTUs. The captive frogs exhibited 15 core OTUs in addition to the above overall core microbiota, whereas the wild frogs exhibited 19 core OTUs in addition to the above overall core microbiota. Predictions made using Phylogenetic Investigation of Communities by Reconstruction of Unobserved States (PICRUSt) suggested that eleven KEGG pathways, such as infectious diseases, immune system diseases, metabolism, metabolism of other amino acids, metabolism of cofactors and vitamins, metabolism of terpenoids and polyketides, neurodegenerative diseases, and transport and catabolism, were enriched in captive frogs. The relative abundance of several red-leg-syndrome-related pathogens increased significantly in captive frogs compared with that in wild frogs. To our knowledge, this is the first study on the effects of individual seasons and captivity on the gut microbiota of frogs.

## Introduction

Vertebrates harbor and interact with diverse microbiotas that influence the physiology of the vertebrate hosts in many ways (e.g., impacting nutrient acquisition and immune responses) and affect their overall health, development, reproduction and behavior (Cryan and Dinan, 2012; Kamada et al., 2013; Lizé et al., 2014). To date, numerous experimental and comparative studies have investigated how factors such as diet, infection status or host phylogeny may impact the gut microbiota (Kohl and Yahn, 2016; Jiménez and Sommer, 2017). Most studies on the gut microbiome have been conducted in mammals, especially laboratory rodents and humans (Kohl and Yahn, 2016), in comparison there is a paucity of information regarding the gut microbiome of other vertebrates such as amphibians.

The gut microbial communities of animals are affected by exogenous and endogenous factors, including ontogenetic stages (Kohl et al., 2013), physiological factors (Dehler et al., 2017), the dietary composition and trophic levels (Liu et al., 2016; Vences et al., 2016), seasonal changes (Weng et al., 2016) and habitat and environmental conditions (Kohl and Yahn, 2016; Longo and Zamudio, 2017). The seasonal variations in the terrestrial habitat conditions of the brown frog *Rana dybowskii*, including variations in temperature and the bacterial flora populations of terrestrial habitats, can be expected to influence the gut microbiota of this frog (Hovda et al., 2012). Furthermore, with seasonal changes in temperate regions, frogs experience changes in food composition and undergo fasting and eating periods. The body temperature of amphibians varies with the temperature of the environment. The frog’s body temperature affects its appetite, metabolism, and gut microbiota (Liu et al., 2016). Amphibians experience many physiological and morphological changes during development. Their diet also changes greatly during development. For instance, tadpoles depend nearly entirely on plant materials, whereas frogs eat mainly insects (Chang et al., 2016). During development from tadpoles to frogs and periodic changes from feeding to fasting, the composition and diversity of gut microbes are significantly altered (Weng et al., 2016). Captive breeding appears to alter the host microbiome. However, few studies have shown the effects of seasonal changes, changes in the growth environment, and individual growth and development on the intestinal microbiota of frogs.

Due to infectious diseases, habitat destruction, climate change, invasive species, and chemical pollutants (Blaustein and Kiesecker, 2002; Jiménez and Sommer, 2017), amphibians are faced with severe population decreases and extinction, highlighting the urgency of conservation (Blaustein and Kiesecker, 2002; Jiménez and Sommer, 2017). Among all wild animal conservation methods, management actions such as the establishment of captive breeding and reintroduction programs have great potential to prevent the extinction of endangered amphibians (Brannelly et al., 2016; Jiménez and Sommer, 2017), but the success of these efforts varies greatly. Captive breeding has largely failed to produce large numbers of viable offspring for release (Kelleher et al., 2018). Studies of the gut microbiota of captive populations may improve this situation, as the important ways in which the gut microbiota may affect amphibian conservation include impacts on host health maintenance, disease relief, improvement of culture conditions, reintroduction success, and invasive species management (Jiménez and Sommer, 2017). The conditions in which amphibians live in captivity are very different from those in the wild, and captive breeding seems to change the host microbiota. For instance, abiotic factors such as temperature, diet composition, and the lack of natural microbiota reservoirs in captive enclosures can modify the gut microbiota of the amphibian host (Becker et al., 2014). Analysis of the effects of changes in host habitat on the skin or intestinal microbiota remains a major research hotspot (Xiang et al., 2018). Several studies have revealed differences in the skin microbiota between captive and wild amphibians (Becker et al., 2014; Loudon et al., 2014; Bataille et al., 2016; Xiang et al., 2018). *R. dybowskii* is a completely terrestrial species with a long breeding cycle, typically 2–3 years, and its rearing methods are very different from those of other frogs of economic importance, such as the bullfrog (Tong et al., 2018). Studying the gut microbiota of *R. dybowskii*, which is a completely terrestrial species that has been domesticated at a high density for 2 years, gives us a rare opportunity to understand the gut flora under such conditions.

*Rana dybowskii* is mainly distributed in northeastern China. This frog is an important species with both medicinal and economic value, and over-capture of this species has led to a significant population decline (Hu et al., 2016). In addition, frequent bacterial infections are responsible for frog diseases and are associated with high overall death rates. Specifically, brown frogs are threatened by infectious diseases such as red-leg syndrome (RLS) (Pasteris et al., 2006; Weng et al., 2016). Breeding and maintaining frogs under captive conditions are likely to affect the gut microbiota of individuals to the detriment of fitness, nutrition, and susceptibility to pathogens. In wild frogs, most health assessments focus on behavioral patterns as well as the psychological and physical conditions of the frogs. Despite the established associations between the gut microbiota and the health status of the host, few studies on the host-associated microbiota of frogs have been performed (Jiménez and Sommer, 2017). If the state of the gut microbiota can be well correlated with the health assessment of captive frogs, it will aid the management of amphibians to establish captive breeding.

The aims of this study are to investigate the effects of season, development and captivity on the intestinal microbiota of *R. dybowskii* and to further explore the differences between potential pathogens of RLS in captive and wild populations. In this study, brown frogs were sampled from a natural distribution zone (wild group) or an intensive culture farm (captive group). The wild frogs were sampled at two development stages: juvenile and adult. The captive brown frogs were sampled in the summer and autumn at a culture farm. Differences in the gut microbiota were compared (1) between juveniles and adults of wild *R. dybowskii* from the same habitat, (2) between the summer and autumn in *R. dybowskii* from the same population in the farming environment, and (3) between captive frogs and wild frogs.

## Materials and Methods

### Animal Habitat Environment

The wild brown frogs were sampled from a natural distribution zone, and they prefer to live in deciduous broad-leaved forests, or coniferous and broad-leaved forests dominated by deciduous broad-leaved forests, and their associated forest edge thickets, forest creeks, and forest swamps. Brown frogs mainly eat insects, followed by earthworms, mollusks and spiders.

The captive brown frogs were sampled from an intensive culture farm. The captive brown frogs derived from wild frog eggs, and wild frog eggs came from the same area as the wild frogs. Wild frog eggs were brought to the farm and hatched, and the tadpoles and young frogs were raised in the captive environment.

The breeding pens were greenhouse facilities enclosed by a sealed fence, equipped with a sprinkler and planted with low vegetation. All pens were made of the same construction materials and displayed the same physical properties. Each pen was approximately 80 m^2^ in area. The ground inside the breeding enclosure consisted of land that was not completely bare and was planted with sparse low vegetation. The air humidity in the pens was generally stable and was controlled at 60–80% by spraying water, and the ground-level humidity of the pens was controlled at 25–35%. The farming density of the 2-year-old frogs was 40/m^2^. Disinfection with iodine volts was conducted two to three times a week. When the average temperature was above 10°C (when the frogs had rested inside the breeding fence for approximately a week) and the frogs were observed to be moving, we provided living yellow mealworms (*Tenebrio molitor*). In the experiment, the frogs were fed two times a day at 09:00 and 16:00, and the total amount fed was approximately 4% of their mean body weight.

Several pathogenic bacteria are associated with RLS, a major infectious disease that causes high mortality in amphibians. The etiological agents include *Aeromonas hydrophila, A. sobria*, *Citrobacter freundii*, *Chryseobacterium indologenes*, *Edwardsiella tarda*, *Proteus mirabilis*, *P. vulgaris*, *Pseudomonas aeruginosa*, *Staphylococcus epidermidis*, and *Streptococcus iniae* (Glorioso et al., 1974; Mauel et al., 2002; Pasteris et al., 2006; Schadich and Cole, 2009; Xie et al., 2009; Zhang et al., 2014; Weng et al., 2016).

### Experimental Design and Sample Collection

Brown frogs of the wild and captive groups were sampled from a natural distribution zone (wild group, W01–W13) and an intensive culture farm (captive group, C01–C12). Wild *R. dybowskii* were captured from the brown frog natural partition of Luobei County (47°65′24″N, 130°46′24″ E, 98 m alt.), Heilongjiang, China. Captive *R. dybowskii* were captured from a frog farm in Huanan County (46°44′54″N, 130°69′32″E; 80 m alt.), Heilongjiang Province, China.

The two sample groups in captive frogs were designated summer group (CS group) and the autumn group (CA group). Captive frogs captured on 5 June and 15 September corresponded to the summer group (CS group) and the autumn group (CA group), respectively. The 2-year-old frogs that were sampled, which were re-fed in captivity after artificial wintering, had been fed for 20 days on 5 June and 120 days on 15 September. The two sample groups were designated CS and CA, and their body weights were 4.65 ± 0.31 g and 22.36 ± 2.65 g, respectively. The samples of the CA (autumn, September 15) group were numbered C01–C07, and the male to female ratio was 3:4. The samples of the CS (summer, June 5) group were numbered C08–C12, and the male to female ratio was 2:3.

The two sample groups of wild frogs were designated the juvenile group (W1) and adult group (W2). The wild frogs captured on June 5 were divided into sexually immature young frogs and sexually mature adults. The samples of the juvenile group (W1) were numbered W01–W06, and the male to female ratio was 3:3. The samples of the adult group (W2) were numbered W07–W13, and the male to female ratio was 3:4. The body weights of the juvenile and adult wild frogs were 5.12 ± 0.57 g and 25.65 ± 3.54 g, respectively. It is difficult to distinguish between males and females in frogs of less than 10 g; anatomical observation of gonad development can be used to distinguish sex as well as sexual maturity. After hibernation, the 2-year-old *R. dybowskii* frogs generally weighed 3–5 g and did not reproduce. All male and female frogs weighing more than 20 g typically pair and spawn after hibernation.

All of the frogs were lively and vigorous. The frogs were first washed with tap water and then with sterilized water. The frogs were examined by intestinal dissection under euthanasia. The contents of the gut samples were collected from the gut within 20 min after euthanasia. We used a fresh pair of sterilized tweezers for the collection of each sample. The digestive tract was then carefully isolated from the body, and a portion of the gut was collected, extending from (but excluding) the stomach to the anus of the digestive tract. The contents of each gut were emptied into a sterile vial and immediately stored at −80°C.

### DNA Extraction and PCR Amplification

Total genomic DNA was extracted from each sample with a FastDNA^®^ Spin Kit for Soil (MP Biomedical, United States) according to the manufacturer’s protocol, and the DNA concentration was measured on a NanoDrop 2000 spectrophotometer (Thermo Fisher Scientific, Wilmington, MA, United States). The V3-V4 hypervariable regions of the bacteria 16S rRNA gene were amplified with primers 338F (5′-ACTCCTACGGGAGGCAGCAG-3′) and 806R (5′-GG ACTACHVGGGTWTCTAAT-3′) (Mori et al., 2014) in a thermocycler PCR system (GeneAmp 9700, ABI, United States). The PCR conditions were as follows: 95°C for 4 min; 27 cycles of 95°C for 30 s, 55°C for 30 s, and 72°C for 45 s; and extension at 72°C for 10 min. The PCR products were separated in agarose gels (2% in Tris-acetate-EDTA buffer) containing ethidium bromide, purified with an AxyPrep DNA Gel Extraction Kit (Axygen Biosciences, Union City, CA, United States) and quantified for DNA content in a QuantiFluor^TM^-ST fluorescence quantitative instrument (Promega, United States) according to the manufacturer’s protocol.

### Illumina MiSeq Sequencing

Purified amplicons were pooled in equimolar amounts subjected to paired-end sequencing (2 × 300) on the Illumina MiSeq platform (Illumina, San Diego, United States) according to standard protocols. The raw reads were deposited in the NCBI Sequence Read Archive (SRA) database (Accession Number: PRJNA422729 (CA group), PRJNA509081 (CS group), PRJNA509086 (W2 group) and PRJNA428920 (W1 group, SAMN08324470- SAMN08324475)].

### Processing of Sequencing Data

Raw fastq files were demultiplexed, quality filtered with Trimmomatic and merged with FLASH according to the following criteria. First, 300 bp reads at any site with an average quality score <20 were truncated across a 50 bp sliding window such that only reads ≥50 bp were retained for analysis. Second, incorrect barcode sequences, sequences with two nucleotide mismatches in the primer, and reads with ambiguous characters were omitted. Third, only sequences with >10 bp of overlap were assembled according to the overlapping sequence, and unassembled reads were omitted. Operational taxonomic units (OTUs) were clustered with a 97% similarity cutoff using UPARSE (version 7.1^1^), and chimeric sequences were identified and removed using UCHIME. The taxonomy of each 16S rRNA gene sequence was analyzed with the RDP Classifier algorithm^2^ against the Silva (SSU128/16 s) 16S rRNA gene database using a confidence threshold of 70% (Cole et al., 2008).

### Statistical Analyses

Alpha diversity indices were compared via the Wilcoxon rank-sum test using Mothur (Schloss et al., 2011), including community diversity indices (Shannon and Simpson), and community richness indices (Chao1 and abundance-based coverage estimator (ACE)]. A Bray–Curtis dissimilarity matrix was calculated based on these profiles and used to perform non-metric multidimensional scaling (NMDS). To test whether the growth stage or growing environment affected community clustering and group dispersion, we modeled weighted UniFrac distances and Bray–Curtis dissimilarities based on an OTU-level table via one-way analysis of similarity (ANOSIM) (Anderson, 2001).

The bacterial taxonomies of the communities were tested using R software. The relative abundance of bacterial phyla and family between the different groups were compared via the Wilcoxon rank-sum test and multiple test correction (Benjamini-Hochberg FDR). A Venn diagram was generated using the R package (version 3.1.0, R Core Team, Auckland, New Zealand) to show unique and shared OTUs. The core gut microbiota of the frogs was assigned if it was found in 90 or 100% of the samples and represented >0.1% of the reads. We used the linear discriminant analysis (LDA) effect size (LEfSe) to identify significant associations between bacterial taxa and different groups (i.e., between Cs and Ca and between C and W). The relative abundance of potential opportunistic pathogens in the gut of brown frogs between the C and W groups was compared via a zero-inflated Gaussian mixture model (metagenomeSeq) using the R stats package and the Python SciPy package (Paulson et al., 2013).

### Predicted Metagenomes

The functional shifts in the microbiotas of the two *Rana* species were predicted using PICRUSt, which can both predict the most recent Kyoto Encyclopedia of Genes and Genomes (KEGG) ortholog (KO) functional profiles of microbial communities via 16S rRNA gene sequences (Langille et al., 2013) and link OTUs with gene contents via a phylogenetic tree of 16S rRNA gene sequences. Thus, the PICRUSt forecast relies on the tree topology, distance to the next organism, and identification of the nearest neighbor, even in the case of large distances. The relative abundance between the different groups were compared via the Wilcoxon rank-sum test and multiple test correction (Benjamini-Hochberg FDR). Only differences with corrected *P-*values < 0.05 are presented.

## Results

### Sequencing Depth, Core Microbiota, and Shared and Unique OTUs

A total of 25 *R. dybowskii* were sampled for sequencing, resulting in 1,147,477 high-quality sequences with a mean of 45,899 sequences per sample. The sequences were classified as OTUs according to >97% sequence identity, and 841 OTUs with an average length of 437.90 bp per read were obtained. The plateau status of the rarefaction curves indicated a sufficient depth of sequencing (Supplementary Figure S1).

**FIGURE 1:**
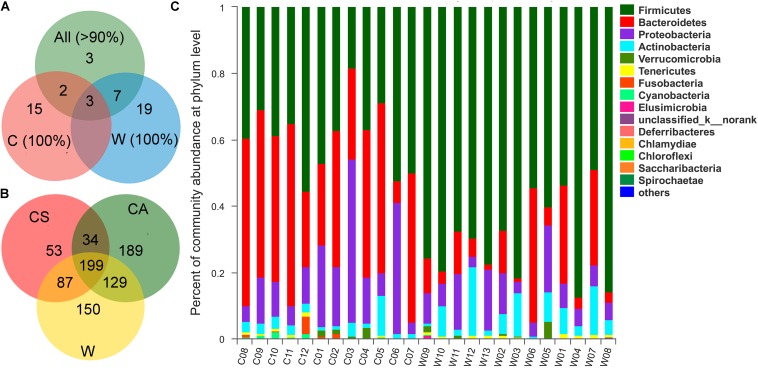
**(A)** Veen diagrams show the number of shared and unique core Operational taxonomic units (OTUs) among the core OTUs of all samples (>90%), the core OTUs of wild samples (100%), and the core OTUs of captive samples (100%). **(B)** Veen diagrams show the number of shared and unique OTUs among CS, CA, and W groups. **(C)** Community bar plot analysis of bacterial at the phylum level.

With respect to the core microbiota in all frogs, the core OTUs that were found in >90% of all frogs tested encompassed 15 core OTUs (Figure 1A). Three of these OTUs (Bacteroidaceae OTU308, Erysipelotrichaceae OTU649, OTU845) existed in all frogs. The core OTUs (found in >90% of all frogs) shared 5 OTUs with those found in captive frogs and 10 OTUs with those of wild frogs. When separated by treatment group, the captive frogs shared a core microbiota of 20 OTUs, including 15 OTUs in addition to the above overall core microbiota, and the wild frogs shared a core microbiota of 29 OTUs, with 19 OTUs in addition to the above overall core microbiota (Figure 1A and Supplementary Table S1).

Most OTUs (415 OTUs) were shared between C (captive frogs, 691 OTUs) and W (wild frogs, 565 OTUs) groups, however, the unique OTUs of the C group was 276, which was more than 150 OTUs of the W group (Figure 1B). The total OTUs of the CS (summer) group was 373, and the OTUs of the CA (autumn) group w2as 551, and shared OTUs between the CS and CA groups was 233 (Figure 1B).

### Differences in Gut Microbiota Between the W1 (Juvenile) and W2 (Adult) Groups in Wild Frogs

**FIGURE 2:**
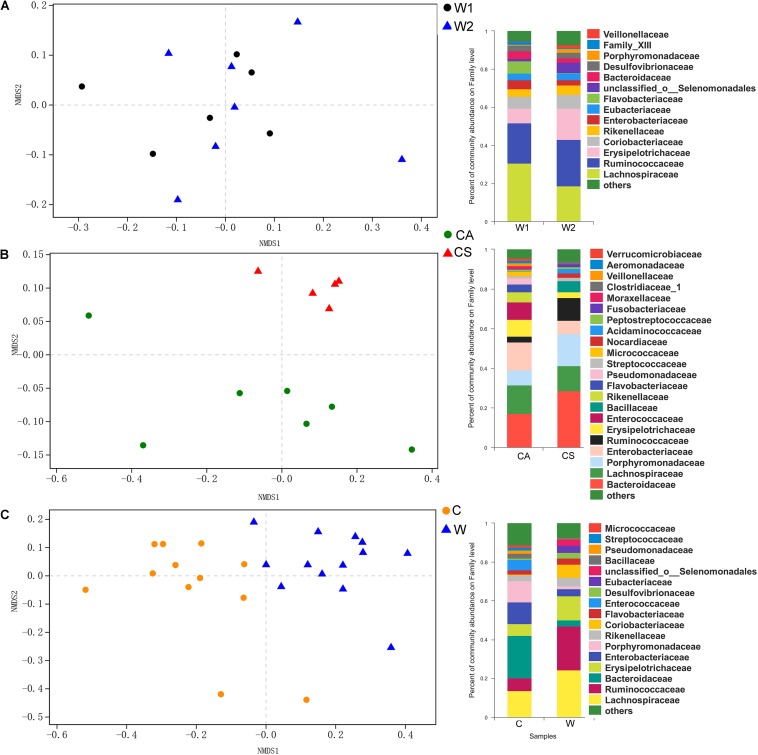
Non-metric multidimensional scaling (NMDS) plot based on the weighted UniFrac distance matrix between the gut bacterial communities of (**A**, left) the W1 (juvenile) and W2 (adult) groups of wild frogs, (**B**, left) the CS (summer) and CA (autumn) groups of captive frogs, and (**C**, left) the C (captive frogs) and W (wild frogs) groups. Composition (at the family level) of the gut bacterial communities of (**A**, right) juvenile and adult wild frogs, (**B**, right) the summer and autumn groups of captive frogs and (**C**, right) captive and wild frogs.

There were no significant differences in richness and diversity between juvenile and adult wild frogs (Kruskal–Wallis rank sum test, *P* > 0.05). Firmicutes (69.9%) was the most dominant bacterial phylum, followed by Bacteroidetes (12.23%), Proteobacteria (9.14%), and Actinobacteria (7.08%) in wild frogs (Figure 1C). The differences in the microbial communities between the samples were visualized in an NMDS plot (Figure 2A), and the results showed that there was no clear segregation between the young frogs and the adult frogs in the wild frog group (Figure 2A). The gut bacterial communities of the juvenile and adult wild frogs did not differ in composition (ANOSIM: Bray–Curtis, *r* = −0.067, *P* = 0.801; weighted UniFrac, *r* = 0.138, *P* = 0.884).

### Differences in Gut Microbiota Between the CS (Summer) and CA (Autumn) Groups of Captive Frogs

There were no significant differences in richness and diversity between the summer and autumn groups (Kruskal–Wallis rank sum test, *P* > 0.05; Table 1). The NMDS plot showed that the bacterial communities significantly segregated into two main groups, one corresponding to the CS group and the other to the CA group (Figure 2B). The gut microbiota composition differed significantly between the summer and autumn groups of captive frogs according to the Bray–Curtis dissimilarity matrix (ANOSIM: Bray–Curtis, *r* = 0.690, *P* = 0.001) but not the weighted UniFrac dissimilarity matrix (ANOSIM: weighted UniFrac, *r* = 0.191, *P* = 0.076).

**TABLE 1:** Alpha-diversity of the brown frog gut microbiota in different groups, as determined by Kruskal–Wallis rank sum test.

**Estimators**	**CS group Mean ± SD**	**CA group Mean ± SD**	**C group Mean ± SD**	**W group Mean ± SD**
Sobs	288.00 ± 12.71^a^	227.86 ± 75.38^a^	252.92 ± 64.17^a^	249.38 ± 41.08^a^
Ace	321.61 ± 17.05^a^	288.28 ± 49.49^a^	302.17 ± 41.67^a^	292.00 ± 44.21^a^
Chao1	324.67 ± 20.77^a^	284.42 ± 59.99^a^	301.19 ± 50.49^a^	299.79 ± 48.03^a^
Shannon	3.34 ± 0.29^a^	3.17 ± 0.74^a^	3.24 ± 0.58^a^	3.48 ± 0.72^a^
Simpson	0.11 ± 0.04^a^	0.11 ± 0.08^a^	0.11 ± 0.06^a^	0.09 ± 0.09^a^

*Different superscripted letters indicate statistically significant differences (*P* < 0.05).*

Taxonomic assignment analysis showed that the most abundant phyla in both the summer and autumn groups were Bacteroidetes (CS: 44.59%, CA: 34.36%), Firmicutes (CS: 39.95%, CA: 38.68%), Proteobacteria (CS: 9.14%, CA: 22.16%), and Actinobacteria (CS: 3.02%, CA: 3.18%) (Figure 1C). There were 16 phyla in captive frogs, and Cyanobacteria, Verrucomicrobia, Tenericutes, and Chlamydiae were found significant differences between the summer and autumn groups (adjusted *P* < 0.05, Wilcoxon rank-sum test and multiple test correction with Benjamini-Hochberg FD). At the family level, Bacteroidetes, Lachnospiraceae, Enterobacteriaceae, Porphyromonadaceae, and Ruminococcaceae were the most abundant bacterial families in both the summer and autumn groups (Figure 2B).

Linear discriminant analysis effect size showed that no phylum was significantly enriched in either the summer or autumn group (LDA > 4, *P* < 0.05, Supplementary Figure S2). LEfSe showed that were Ruminococcaceae, Porphyromonadaceae, Bacteroidaceae, Acidaminococcaceae and Nocardiaceae significantly enriched in the summer group, whereas Rikenellaceae, Erysipelotrichaceae, Flavobacteriaceae, and Enterococcaceae were significantly enriched in the autumn group (LDA > 4, *P* < 0.05, Supplementary Figure S2).

Linear discriminant analysis effect size analysis of the microbiota of captive frogs at the family level indicated that 42 of the 123 families present in the dataset were differentially abundant between the summer and autumn groups (LDA > 2, *P* < 0.05). Among these 42 families, 30 were more abundant in the summer group, and 12 were more abundant in the autumn group (Supplementary Table S2). In addition, 88 of the 280 genera and 123 of the 432 species present in the dataset differed in relative abundance between the summer and autumn groups. Among the group of 88 genera, 61 and 27 genera were more abundant in the summer and autumn groups, respectively, while among the 123 species, 90 and 33 were more abundant in the summer and autumn groups, respectively (LDA > 2, *P* < 0.05) (Supplementary Tables S3, S4).

**FIGURE 3:**
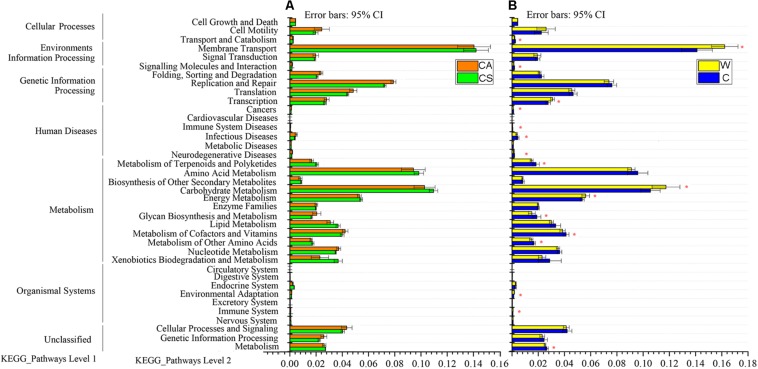
Relative abundances of predicted genes in the metagenome related to level-1 and level-2 KEGG pathways; **(A)** red boxes, CS (summer) group; green boxes, CA (autumn) group; **(B)** yellow boxes, W (wild frogs) group; blue boxes, C (captive frogs) group. Asterisks indicate significant differences between groups.

Predicted functional analysis of the gut microbiota of the summer and autumn groups by PICRUSt revealed 39 level-2 KO groups, as shown in Figure 3. There was no significant enrichment of KEGG pathway in summer group or autumn group (adjusted *P* > 0.05, Wilcoxon rank-sum test and multiple test correction with Benjamini-Hochberg FD, Figure 3A).

### Differences in Gut Microbiota Between the C (Captive Frogs) and W (Wild Frogs) Groups

There were no significant differences in richness or diversity between the wild and captive groups (Kruskal–Wallis rank sum test, *P* > 0.05; Table 1). The NMDS analysis demonstrated that the bacterial communities from the wild and captive frogs significantly segregate into two main groups (Figure 2C). The gut bacterial communities of captive and wild frogs differed in both composition and structure (ANOSIM: Bray–Curtis, *r* = 0.569, *P* = 0.001; weighted UniFrac *r* = 0.632, *P* = 0.001).

Of all 21 phyla, seven of these bacterial phyla, such as Firmicutes, Bacteroidetes, Fusobacteria, Elusimicrobia, Deferribacteres, and Saccharibacteria, exhibited significant differences between the wild and farmed groups (adjusted *P* < 0.05, Wilcoxon rank-sum test and multiple test correction with Benjamini-Hochberg FD). At the family level, Bacteroidetes (21.87 vs. 3.12% in wild frogs) and Lachnospiraceae (13.66 vs. 24.21% in wild frogs) were the most abundant families in captive frogs, and Lachnospiraceae (24.21 vs. 13.66% in captive frogs) and Ruminococcaceae (22.84 vs. 6.46% in captive frogs) were the most abundant families in wild frogs (Figure 2C).

Eleven KEGG pathways, such as cancers, glycan biosynthesis and metabolism, infectious diseases, immune system diseases, metabolism, metabolism of other amino acids, metabolism of cofactors and vitamins, metabolism of terpenoids and polyketides, neurodegenerative diseases, signaling molecules and interaction, and transport and catabolism, were significantly enriched in captive frogs. Six KEGG pathways (cardiovascular diseases, environmental adaptation, energy metabolism, immune system, membrane transport carbohydrate metabolism, and transcription) were significantly enriched in wild frogs (adjusted *P* < 0.05, Wilcoxon rank-sum test and multiple test correction with Benjamini-Hochberg FD, Figure 3B).

**FIGURE 4:**
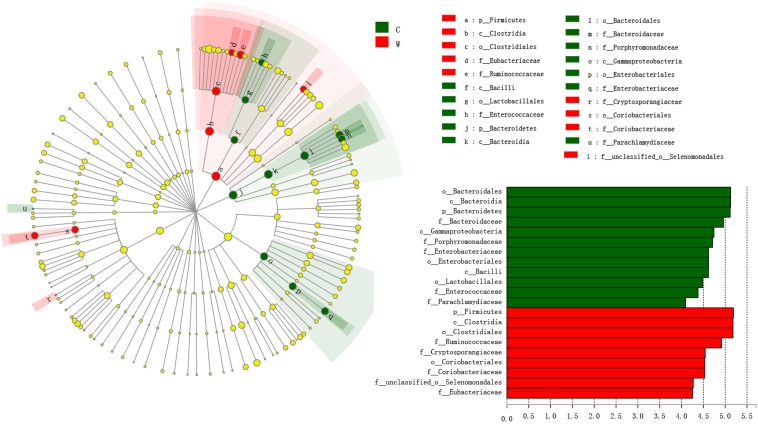
Family, order, class and phyla that were differentially represented between captive groups and wild groups, as indicated by relative abundances based on linear discriminant analysis effect size (LEfSe) analysis linear discriminant analysis (LDA > 4, *P* < 0.05). Red boxes and green boxes represent enrichment in the W (wild) and C (captive) groups, respectively. Each circle’s diameter is proportional to the taxon’s abundance. The strategy of multiclass analysis is non-strict (at least one class differential). Circles represent taxonomic ranks from domain to family, from the inside to the outside. Circles represent taxonomic ranks from phylum to genus, from the inside to the outside. Labels are shown at the class, order and family levels. The scores are shown for all taxa with an LDA score > 4.

Linear discriminant effect size analysis showed that Bacteroidetes and Firmicutes were significantly enriched in captive and wild frogs, respectively (LDA > 4, *P* < 0.05, Figure 4). LEfSe analysis showed that Bacteroidaceae, Parachlamydiaceae, Porphyromonadaceae, Enterobacteriaceae, and Enterococcaceae were significantly enriched in captive frogs, whereas Ruminococcaceae, Cryptosporangiaceae, Coriobacteriaceae, Eubacteriaceae and an unclassified family in order Selenomonadales were significantly enriched in wild frogs (LDA > 4, *P* < 0.05, Figure 4).

Linear discriminant effect size analysis at the family level revealed that 26 of 123 families were clearly more abundant in captive frogs and that 21 were more abundant in wild frogs (Supplementary Table S5). In addition, 113 of 280 genera and 156 of 432 species present in the dataset differed in relative abundance between the C and W groups (LDA > 2, *P* < 0.05). Among the 113 detected genera, 55 and 58 were more abundant in captive and wild frogs, respectively, while amonvg the 156 species, 79 and 77 were more abundant in captive and wild frogs, respectively (Supplementary Tables S6, S7).

The differential abundance of OTUs among treatments was evaluated with metagenomeSeq, and we found that the genus *Citrobacter*, *Pseudomonas*, *Staphylococcus*, and *Streptococcus* were significantly higher in relative abundance in captive frogs than wild frogs (adjusted *P*-value < 0.05; Table 2), however, the genera *Proteus*, *Staphylococcus*, and *Streptococcus* were not characterized in wild frogs (Table 2). Other RLS-related pathogens, such as *Edwardsiella*, were not characterized in all frogs.

**TABLE 2:** Mean relative abundances of potential opportunistic pathogens in the guts of brown frogs, as determined by the by metagenomeSeq with *P*-value and adj *P*-values.

**Species name**	**C group Mean ± SD (%)**	**W group Mean ± SD (%)**	***P*-value**	**Adj *P*-values**
*Aeromonas*	0.75 ± 1.63	0.20 ± 0.37	0.9556	0.9628
*Citrobacter*	9.89 ± 9.42	3.14 ± 5.42	0.0050	0.0275
*Chryseobacterium*	0.20 ± 0.39	2.92 ± 10.36	0.4613	0.5412
*Proteus*	0.01 ± 0.01	0	0.0820	0.1658
*Pseudomonas*	1.84 ± 3.05	0.06 ± 0.09	0.0080	0.0354
*Staphylococcus*	0.01 ± 0.01	0	0.0264	0.0412
*Streptococcus*	0.05 ± 0.07	0	0.0019	0.0185

## Discussion

### Effects of Developmental Stage on the Gut Microbiota of Wild *R. dybowskii*

The gut microbial communities of wild frogs were similar in composition between the different developmental stages in frogs in the same habitats and with similar food compositions, or under the regulation of similar physiological processes. Although previous research has confirmed that bacterial communities change during host development and growth (Hu et al., 2018), some studies have shown that bacterial communities are similar between the juvenile and adult stages of certain animal hosts (Xue et al., 2015).

The juvenile and adult wild frogs included in this study were collected from the same site. *R. dybowskii* is a fully land-dependent species that lives in deciduous broad-leaved forests or mixed broadleaf-conifer forests dominated by deciduous broad-leaved forests as well as among suitable forest-margin shrubs, along within-forest creeks, or in within-creek swamps (Xu et al., 2010). *R. dybowskii* catches only live prey and unselectively attacks any food available, with a largely consistent diet composition being observed between juveniles and adults. In addition, the juvenile and adult brown frogs that we collected all had well-developed digestive systems; thus, the diet was likely highly similar between the juvenile and adult groups.

The main differences between the two sampled populations were gonadal development, body size, and growth rate. These factors may have influenced the gut bacterial composition, and due to differences in colonization conditions, bacteria in the more mature brown frogs required a longer time for colonization. However, both juvenile and adult frogs undergo a long hibernation; remaining in low-temperature waters and fasting for 6 months causes the hibernating frogs to lose weight and to consume a large amount of endogenous fats (Cramp and Franklin, 2005). Thus, immediately upon taking in food, juvenile and adult frogs display similar physiological demands *in vivo* (Gavira et al., 2018). The sampling time in this study was approximately 20 days after the onset of food intake. Despite size differences among individuals, the guts of the frogs can be expected to adjust similarly from the hibernation-adapted microbiota to the feeding-adapted microbiota. Therefore, in hibernating *R. dybowskii*, the intense effects of periodic fasting and feeding may be expected to dominate other weaker factors, leading to a similar gut microbiota between juvenile and adult frogs.

### Effects of Season on the Gut Microbiota of Captive *R. dybowskii*

In early June and September, frogs were bred in the same breeding field, and the breeding management measures and procedures were largely consistent. These two groups experienced consistent environments and were fed the same food, *T. molitor*. However, the average temperature of the breeding pens in early summer (June 5) was significantly higher than that in autumn (September 15) (Qing et al., 2012). Temperature significantly impacts the community structure and membership of the frog gut (Kohl and Yahn, 2016). In addition, the microbiota required a longer time to undergo permanent implantation in the gut in the autumn group because the frogs in the summer and autumn groups were sampled 20 days and 120 days after starting to eat, respectively. Furthermore, the food demands of the two groups were different. Soon after hibernation, the frogs actively consumed food, entered the fast-growing stage and required more nutrients than they did during the growth stage (Tong et al., 2018). The frogs consumed food less actively in September than in the summer. These different physiological states are reflected in the corresponding gut microbiota of the frogs.

### Differences in Living Environment Between Wild and Captive Frogs

Consistent with other studies of *Neophoca cinerea*, *Grus japonensis*, *Salmo salar* L. and *Paralichthys olivaceus*, intensive culturing has been shown to significantly affected the gut microbiota composition in *R. dybowskii* (Xie et al., 2016; Dehler et al., 2017). We analyzed the factors affecting the gut microbiota of wild and captive brown frogs. Diet, environmental factors, and population density may be important factors. Population density is an important factor affecting the growth and health of wild or captive frogs (Berven, 2009). Frogs living in high-density environments are subject to a suite of environmental conditions that, either alone or in combination, affect growth and health. A high culture density causes the body to produce a stress reaction, which results in changes in neuroendocrine activity, physiological biochemistry and the immune system (De las Heras et al., 2015). These changes in physiological biochemistry and the immune system lead to changes in the gut microbiota. A high farming density also increases competition for living space and food. Changes in animal feed intake may also cause changes in the gut microbiota of the host (Guardia et al., 2011). In addition to the direct stress of a high population density during cultivation, residual feed, feces and urine can transmit toxic and harmful substances (e.g., ammonia and hydrogen sulfide) that are adsorbed by the mucous membranes of animals, causing irritation and inflammation (Kim et al., 2005). Ammonia is considered the most harmful gas in animal pens. Gastroenteritis is associated with high concentrations of ammonia (Ali and Al Swayeh, 2003).

The effect of the food composition on the gut microbiota is pronounced. Brown frogs feed on live food, so farms must supply frogs with live insects, such as *T. molitor*. The diet of the brown frog in the wild includes a variety of animals. Food resources can also change the gut and skin microbiota of amphibian hosts (Antwis et al., 2014; Jiménez and Sommer, 2017). For red-eyed tree frogs (*Agalychnis callidryas*), a carotenoid-enriched diet, rather than a carotenoid-free diet, improves the species richness and abundance of skin bacteria (Antwis et al., 2014). Compared with a highly diverse diet, a less diverse diet reduces the gut bacterial richness of tadpoles of *Boophis reticulatus*, *B. marojezensis*, and *B. narinsi* (subgenus *Chonomantis*).

The environments in which captive and wild brown frogs live vary greatly. The differences in frog gut microbiota composition and structure during culture management can be attributed to changes in environmental factors such as food, humidity, temperature, illumination, antibiotic pollution, and the soil flora (Meron et al., 2011; Becker et al., 2014). There were very few OTUs in the core microbiota (>90%), and only three OTUs existed in all frogs (Figure 1A). This may be related to differences in food and feeding habits. There is only one food (here, *T. molitor*) for cultured frogs, and the diet (T. molitor) was not sequenced along with the frog, while wild frogs have dozens of kinds of food. Captive frogs live on ground (soil) without vegetation, and food is placed on the ground. When captive frogs ingest *T. molitor* in their habitat, their gut microbiota is affected by the soil flora. In contrast, wild frogs capture more flying insects; therefore, their gut microbiota is less affected by the soil flora. The variations in the gut microbiota or core microbiota of captive frogs may be due to exposure to antibiotics (Kueneman et al., 2014). In this study, brown frogs did not receive antibiotics during the experiment, however, the *T. molitor* insects used in this study were purchased from a culture factory, which might use antibiotics to facilitate high-density culturing and prevent diseases. Captive frogs may be prophylactically administered antibiotics to protect against diseases, and exposure to antibiotics may change the relative species abundances of gut microbiota in the long term (Helmick et al., 2018). Such changes can be observed in the gut microbiota of humans after antibiotic treatment (Modi et al., 2014).

### Compositional Differences in the Gut Microbiota Between Wild and Captive Frogs

The three most abundant gut microbiota phyla were Firmicutes, Bacteroides and Proteobacteria in both the captive and wild groups, consistent with findings in related studies of mammals (Nelson, 2015), birds (Wei et al., 2013), *Fejervarya multistriata* (Chang et al., 2016), *R. pipiens* (Kohl et al., 2013) and *Litoria ewingii* (Weng et al., 2016). Firmicutes and Bacteroidetes were the dominant phyla in the gut microbiota of both wild and captive brown frogs, but the Firmicutes/Bacteroidetes ratio was much higher in wild frogs than in captive frogs (69.9/12.2 vs. 39.2/38.6%). This finding is consistent with a previous study that showed that the intestinal Firmicutes/Bacteroidetes ratio in *F. multistriata* was higher in a farmland environment than in a natural living environment (Chang et al., 2016). These differences could be due to the limited food in the harsher, natural environment, in contrast to the abundant food present in farmland environments and our captive environment (Chang et al., 2016). According to previous studies, a higher Firmicutes/Bacteroidetes ratio indicates a higher efficiency of energy uptake from foods (Shortt et al., 2017), which improves the probability of survival.

Firmicutes is critical for carbohydrate fermentation and nutrient absorption (Ramakrishna, 2013), and the intestinal enrichment of this group in *R. dybowskii* may be associated with the food composition during the terrestrial living period. *R. dybowskii* frogs are omnivorous and indiscriminately capture any food that they can obtain; they survive on various insects with species-specific times of appearance. *R. dybowskii* frogs prefer large prey, especially high-protein insects. However, captive brown frogs are fed only *T. molitor*, which is rich in water but lacking in dry matter, such as proteins. Accordingly, the proportion of Firmicutes varies significantly with the food composition (Beloshapka et al., 2013), which affects the intestinal microbiota composition.

Bacteroidetes was the second most dominant gut microbial phylum after Firmicutes in *R. dybowskii* and is critical for the health of brown frogs. *Bacteroides* has probiotic effects on animals by contributing to polysaccharide degradation, nutritional use acceleration, faster intestinal mucosal vascularization, immune system development, host immunity, and gut microbalance preservation (Bäckhed et al., 2004). Moreover, *Bacteroides* is beneficial for polysaccharide utilization by animals (Nathan and Eduardo, 2017). Brown frogs living in harsh environments catch any accessible food, and *Bacteroides* helps *R. dybowskii* adjust to continuously changing food types and assists in polysaccharide digestion in the host by utilizing food particles, the gut mucilage layer, or gut epithelial cells as a source of nutrition (Bäckhed et al., 2005). When a nutritional source is no longer available due to environmental changes, members of *Bacteroides* can adjust their genomes to produce proteins and enzymes, thereby protecting their metabolism and that of their hosts (Nathan and Eduardo, 2017).

Proteobacteria and Actinobacteria, with average relative abundances of 12.79 and 5.18%, respectively, were the richest phyla in the *R. dybowskii* gut microbiota. These phyla strongly affect the normal microbiota composition. Proteobacteria can flexibly adjust metabolism and tolerate low-nutrition foods, and they are more competitive than other microbes (Berg et al., 2016). However, many Proteobacteria genera are conditional pathogens. For instance, *Acinetobacter*, *Pseudomonas* and *Citrobacter*, which were relatively abundant in our study, can induce many diseases in humans and amphibians (Kamei et al., 2010; Raczynski et al., 2012; Gauthier, 2015).

When grown to an appropriate number, probiotics can benefit living microbes of the host (Angahar, 2016). Probiotics have been shown to improve weight gain, feed conversion and apparent survival in bullfrogs (Dias et al., 2010). In this study, *Bacillus* (1.14%) was detected in both captive and wild frogs. Many *Bacillus* species (e.g., *B. coagulans*, *B. mesentericus*, and *B. subtilis*) that were previously confirmed as potential probiotics were unclassified in our study, and their presence should be confirmed in the future. To date, there have been few reports on the effects of *Bacillus* in frog culture, and it is difficult to deem *Bacillus* an effective probiotic, due to the high proportions of *Bacillus* in the guts of frogs.

Our results regarding RLS-related pathogens suggest that high-density breeding may result in the exposure of frogs to potential pathogens, leading to disease. RLS is an infectious disease caused by septicemia and is the leading cause of death in brown frogs. Pathogens that increase the risk of RLS include *A. hydrophila*, *C. indologenes*, *C. meningosepticum*, *C. freundii*, *P. mirabilis*, and *P. aeruginosa* (Zhang et al., 2014). These species are opportunistic pathogens that are normally found in the skin and gut microbiota of healthy frogs (Schadich and Cole, 2009). However, RLS-related pathogens in brown frogs remain uncharacterized. Here, we found that the relative abundances of a few RLS-related pathogenic genera reported in frogs were higher in captive frogs than in wild frogs. For example, the genera *Citrobacter*, *Pseudomonas*, *Staphylococcus*, and *Streptococcus* displayed higher relative abundances in captive frogs than in wild frogs. Other RLS-related pathogens, such as *Edwardsiella*, were not characterized in any of the frogs, while the genera *Proteus*, *Staphylococcus*, and *Streptococcus* were not characterized in the wild frogs (Table 2). Our results may explain the higher mortality of frogs at higher densities in culture environments (Browne et al., 2003).

Overall, significant differences in gut microbiota composition were demonstrated between captive and wild brown frogs; there were also significant differences in the gut microbiota of captive frogs between the summer and autumn. The relative abundances of RLS-related pathogens were significantly higher in captive brown frogs than in wild brown frogs. This study investigated the effects of short-term growth and development on the gut microbiota of *R. dybowskii*; however, because amphibians have complex life histories, it is necessary to further study the changes in the gut microbiota during each stage of growth and development.

## Ethics Statement

All animal protocols were approved by the Institutional Animal Care and Use Committee of Northeast Agricultural University (IACUC#2015-035). All experiments were performed in accordance with approved guidelines and regulations.

## Author Contributions

QT, JB, ZH, and XL: data collection, data analysis and interpretation, and drafting of the manuscript. QT, HW, and JZ: conception or design of the work. QT, JD, and XL: sample collection. QT and ZH: writing and critical revision of the manuscript. JZ: final approval of the version submitted.

## Conflict of Interest Statement

The authors declare that the research was conducted in the absence of any commercial or financial relationships that could be construed as a potential conflict of interest.

## Footnotes

1 http://drive5.com/uparse/

2 https://sourceforge.net/projects/rdp-classifier/
