# Supplementary material for: Effects of Captivity and Season on the Gut Microbiota of the Brown Frog (Rana dybowskii)
Source: Front Microbiol. 2019 Aug 23;10:1912. doi: 10.3389/fmicb.2019.01912 (PMC6716059; doi:10.3389/fmicb.2019.01912)
Supplement: Supplementary file 1 [file Table_1.DOCX]

**Table S1** The core OTUs that were found in > 90% of all frogs, and the wild frogs shared a core microbiota of 29 OTUs, and the captive frogs shared a core microbiota of 20 OTUs.

| Groups | Phylum | Family | OTU |
| --- | --- | --- | --- |
| 1 | Firmicutes | Ruminococcaceae | OTU544 |
| 1 | Firmicutes | Eubacteriaceae | OTU811 |
| 1 | Firmicutes | Lachnospiraceae | OTU468 |
| 1 | Firmicutes | Erysipelotrichaceae | OTU260 |
| 1 | Firmicutes | Erysipelotrichaceae | OTU675 |
| 1 | Bacteroidetes | Bacteroidaceae | OTU308 |
| 1 | Proteobacteria | Enterobacteriaceae | OTU66 |
| 1 | Actinobacteria | Nocardiaceae | OTU817 |
| 1 | Firmicutes | Lachnospiraceae | OTU283 |
| 1 | Firmicutes | Erysipelotrichaceae | OTU649 |
| 1 | Firmicutes | Erysipelotrichaceae | OTU845 |
| 1 | Bacteroidetes | Bacteroidaceae | OTU80 |
| 1 | Proteobacteria | Enterobacteriaceae | OTU813 |
| 1 | Firmicutes | Lachnospiraceae | OTU467 |
| 1 | Proteobacteria | Moraxellaceae | OTU53 |
| 2 | Firmicutes | Ruminococcaceae | OTU533 |
| 2 | Bacteroidetes | Bacteroidaceae | OTU74 |
| 2 | Firmicutes | Ruminococcaceae | OTU544 |
| 2 | Firmicutes | Eubacteriaceae | OTU811 |
| 2 | Firmicutes | Lachnospiraceae | OTU468 |
| 2 | Firmicutes | Lachnospiraceae | OTU763 |
| 2 | Firmicutes | Lachnospiraceae | OTU535 |
| 2 | Firmicutes | Erysipelotrichaceae | OTU675 |
| 2 | Firmicutes | Ruminococcaceae | OTU387 |
| 2 | Bacteroidetes | Bacteroidaceae | OTU308 |
| 2 | Bacteroidetes | Rikenellaceae | OTU630 |
| 2 | Proteobacteria | Enterobacteriaceae | OTU66 |
| 2 | Firmicutes | Ruminococcaceae | OTU714 |
| 2 | Firmicutes | Ruminococcaceae | OTU640 |
| 2 | Firmicutes | Lachnospiraceae | OTU283 |
| 2 | Firmicutes | Lachnospiraceae | OTU561 |
| 2 | Firmicutes | Lachnospiraceae | OTU498 |
| 2 | Firmicutes | Ruminococcaceae | OTU755 |
| 2 | Firmicutes | Lachnospiraceae | OTU385 |
| 2 | Firmicutes | Erysipelotrichaceae | OTU667 |
| 2 | Firmicutes | Erysipelotrichaceae | OTU649 |
| 2 | Firmicutes | Erysipelotrichaceae | OTU845 |
| 2 | Firmicutes | Lachnospiraceae | OTU300 |
| 2 | Firmicutes | Ruminococcaceae | OTU557 |
| 2 | Firmicutes | Ruminococcaceae | OTU801 |
| 2 | Firmicutes | Ruminococcaceae | OTU540 |
| 2 | Firmicutes | Lachnospiraceae | OTU467 |
| 2 | Firmicutes | Lachnospiraceae | OTU520 |
| 2 | Firmicutes | Ruminococcaceae | OTU591 |
| 3 | Firmicutes | Erysipelotrichaceae | OTU260 |
| 3 | Bacteroidetes | Bacteroidaceae | OTU2 |
| 3 | Firmicutes | Erysipelotrichaceae | OTU39 |
| 3 | Proteobacteria | Enterobacteriaceae | OTU1 |
| 3 | Bacteroidetes | Bacteroidaceae | OTU308 |
| 3 | Actinobacteria | Microbacteriaceae | OTU373 |
| 3 | Bacteroidetes | Bacteroidaceae | OTU10 |
| 3 | Actinobacteria | Nocardiaceae | OTU817 |
| 3 | Proteobacteria | Enterobacteriaceae | OTU60 |
| 3 | Actinobacteria | Microbacteriaceae | OTU280 |
| 3 | Firmicutes | Erysipelotrichaceae | OTU649 |
| 3 | Firmicutes | Erysipelotrichaceae | OTU845 |
| 3 | Bacteroidetes | Bacteroidaceae | OTU80 |
| 3 | Bacteroidetes | Bacteroidaceae | OTU530 |
| 3 | Firmicutes | Streptococcaceae | OTU63 |
| 3 | Proteobacteria | Enterobacteriaceae | OTU813 |
| 3 | Firmicutes | Enterococcaceae | OTU732 |
| 3 | Bacteroidetes | Bacteroidaceae | OTU272 |
| 3 | Bacteroidetes | Bacteroidaceae | OTU472 |
| 3 | Bacteroidaceae | Bacteroidaceae | OTU266 |

1 of the first column in the table is a sample of all frogs, and 2 is a sample of wild frogs, and 3 is a sample of captive frogs.
