# Supplementary material for: Effects of Captivity and Season on the Gut Microbiota of the Brown Frog (Rana dybowskii)
Source: Front Microbiol. 2019 Aug 23;10:1912. doi: 10.3389/fmicb.2019.01912 (PMC6716059; doi:10.3389/fmicb.2019.01912)
Supplement: Supplementary file 8 [file Table_8.DOCX]

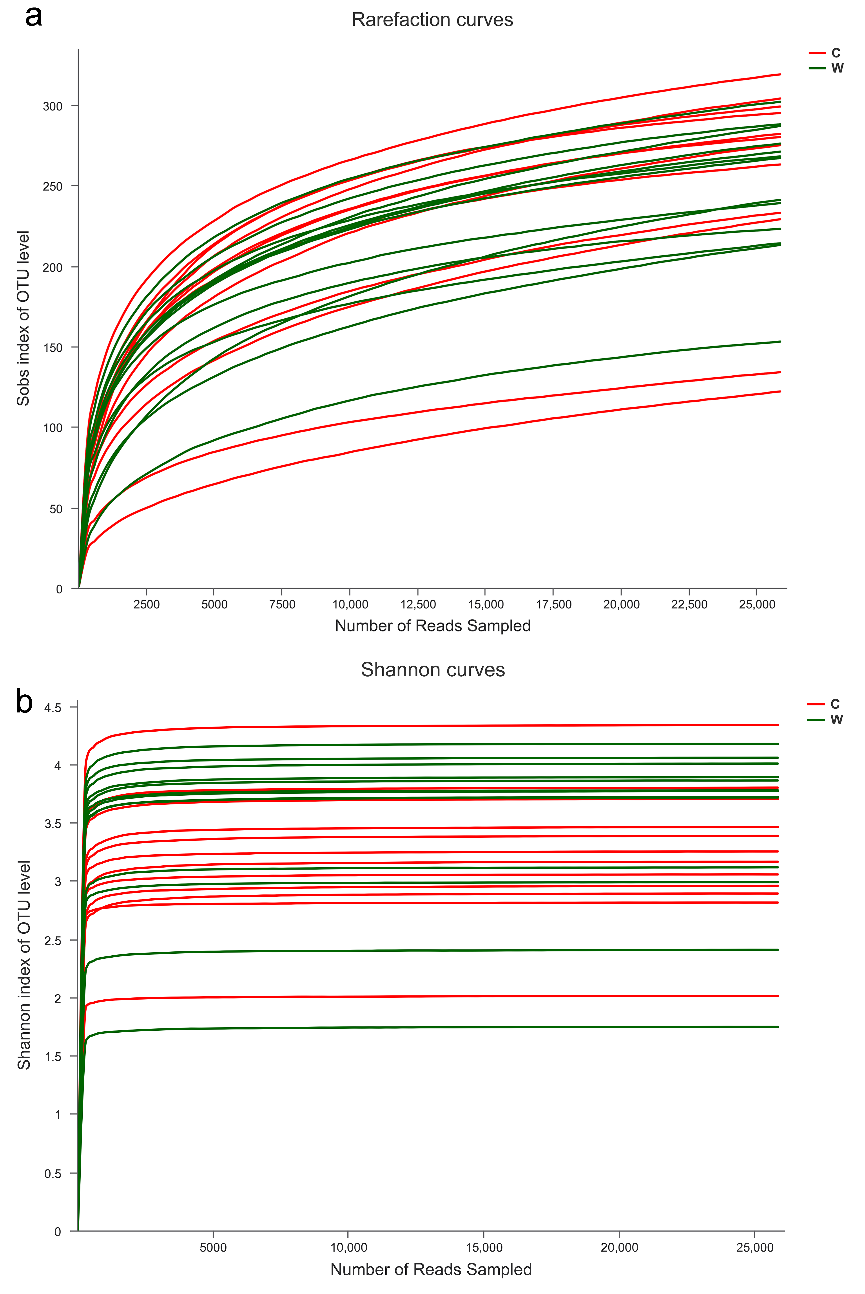


**Figure S1** (a) Rarefaction curves and (b) Shannon curves.


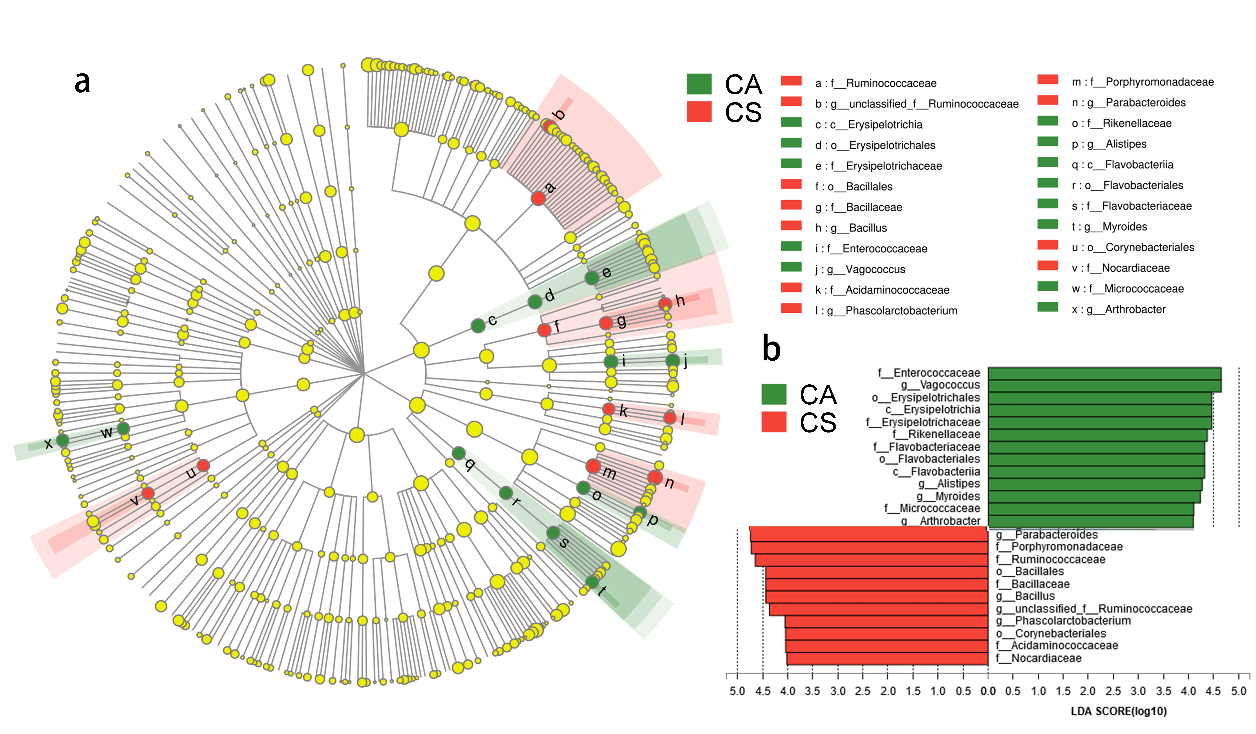


**Figure S2** Cladogram indicating the phylogenetic distribution of microbial lineages associated with the difference in juvenile and adult captive frogs using the linear discriminant analysis (LDA) effect size (LEfSe) method. Differences are represented by treatment colour (red indicating autumn samples (CA), green indicating summer samples (CS). Each circle's diameter is proportional to the taxon's abundance. The strategy of multiclass analysis is non-strict (at least one class differential). Circles represent taxonomic ranks from domain to genus inside to out. Circles represent taxonomic ranks from phylum to genus inside to out. Labels are shown of the class, order and family levels. The scores for all the taxa with LDA score > 4.
